# Red blood cell dynamics in biomimetic microfluidic networks of pulmonary alveolar capillaries

**DOI:** 10.1063/1.4973930

**Published:** 2017-01-10

**Authors:** Hagit Stauber, Dan Waisman, Netanel Korin, Josué Sznitman

**Affiliations:** 1 Department of Biomedical Engineering, Technion – Israel Institute of Technology, 3200003 Haifa, Israel; 2 Department of Neonatology, Carmel Medical Center, 3436212 Haifa, Israel; 3 Faculty of Medicine, Technion – Israel Institute of Technology, 3200003 Haifa, Israel

## Abstract

The pulmonary capillary networks (PCNs) embody organ-specific microvasculatures, where blood vessels form dense meshes that maximize the surface area available for gas exchange in the lungs. With characteristic capillary lengths and diameters similar to the size of red blood cells (RBCs), seminal descriptions coined the term "sheet flow" nearly half a century ago to differentiate PCNs from the usual notion of Poiseuille flow in long straight tubes. Here, we revisit in true-scale experiments the original “sheet flow” model and devise for the first time biomimetic microfluidic platforms of organ-specific PCN structures perfused with RBC suspensions at near-physiological hematocrit levels. By implementing RBC tracking velocimetry, our measurements reveal a wide range of heterogonous RBC pathways that coexist synchronously within the PCN; a phenomenon that persists across the broad range of pressure drops and capillary segment sizes investigated. Interestingly, in spite of the intrinsic complexity of the PCN structure and the heterogeneity in RBC dynamics observed at the microscale, the macroscale bulk flow rate versus pressure drop relationship retains its linearity, where the hydrodynamic resistance of the PCN is to a first order captured by the characteristic capillary segment size. To the best of our knowledge, our *in vitro* efforts constitute a first, yet significant, step in exploring systematically the transport dynamics of blood in morphologically inspired capillary networks.

## I. INTRODUCTION

The past decades have witnessed tremendous efforts to quantify red blood cell (RBC) flows in the microvasculature.^1–12^ Both *in vivo*^13–16^ and *in vitro*^17–19^ studies have been critical in elucidating how microcirculation is highly dynamic^2,3,20,21^ with phenomena that include the Fahraeus effect,^1,2^ the Fahraeus-Lindqvist effect,^3^ plasma skimming,^5^ cell screening,^6^ and the pathway effect^8,12^ amongst other. Such transport dynamics are driven by morphology^4,12^ and size^3,22^ of the microvasculature, as well as local blood viscosity properties due to phase separation,^8,12^ apparent hematocrit^22,23^ (Hct), and changes in mechanical properties of RBCs under diseased conditions.^24^

Amid the singularities of the microvasculature, the high deformability^24–26^ of RBCs enables such cells to travel through capillary vessels where characteristic diameters are similar to, if not smaller than, the actual size of RBCs^11,27^ (∼6–8 *μ*m). In this context, microfluidics has provided an attractive gateway to study at true scale RBC flows *in vitro*.^26,28–31^ Yet, past studies mimicking the microvasculature have been frequently limited to straight channels,^17,32,33^ single vessel branches,^21,34,35^ or bifurcating networks^26,28,36^ where perfusion is often operated under diluted Hct levels.^34,37–40^ Such geometries represent only a small fraction of the rich microvascular diversity intrinsic to the body; microcirculation is generally organ-specific^41–44^ (e.g., mesentery, brain, kidney, liver, retina), where the local vascular morphology diverges significantly from long, straight vessels or simple dichotomous pathways.

**FIG. 1.**
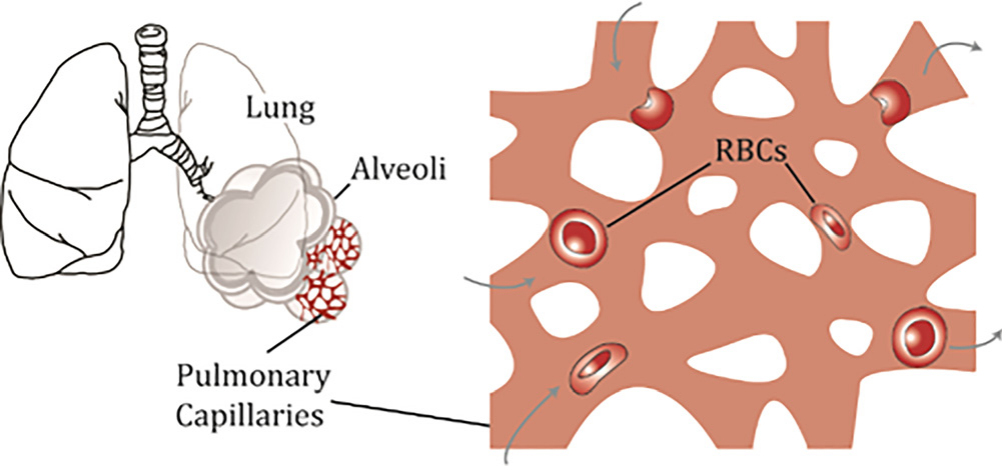
Schematic of the lungs and their terminal units, i.e., the alveolar sacs. The inter-alveolar wall septa contain a dense mesh of pulmonary capillaries that wrap entirely around the alveoli. Inset: schematic illustration of a small region within the pulmonary capillary networks (PCNs), constructed of short individual capillary segments with lengths and diameters similar to the size of individual RBCs.

Pulmonary capillary networks (PCNs) represent a prime example of organ-specific microvasculatures^45,46^ that wrap around the smallest units of the lungs, i.e., the pulmonary alveoli (see schematic of Fig. 1). There, capillaries form close-knit “meshes” to maximize the surface area available for gas exchange between air and blood (Fig. 1, inset). Since individual capillary segments are short and exhibit lengths and diameters of nearly the same size^47^ (∼3–10 *μ*m), seminal descriptions^48^ coined the term "sheet flow" to capture the assembly of such capillaries into quasi-2D networks constructed by the voids formed between regularly positioned posts that fill the available inter-septal alveolar wall. In turn, this specific morphology distinguishes PCNs from the usual notion of Poiseuille flow in long cylindrical tubes. Due to technical limitations at the time, early investigations of “sheet flow” were limited to scaled-up experiments using single-phase Newtonian fluids (e.g., plasma), and thus came short of capturing the anticipated dynamics of real RBCs in such micro-networks. While recent microfluidic efforts have featured lab-on-chip devices exhibiting geometries somewhat similar to those seen in PCNs, these have been geared towards cell sorting applications based on deterministic lateral displacement assays,^31^ rather than motivated by physiological investigations of RBC flows. In the absence of physiologically faithful *in vitro* setups, several numerical studies have attempted to bridge our understanding of RBC transport in capillary networks. Nevertheless, these efforts have either focused on a confined region of capillary flow in the PCN^49–51^ or omitted entirely the role of RBC deformability.^52–54^ Importantly, past simulations have been supported by few, if any, experimental data. To the best of our knowledge, there are currently no experiments quantifying physiologically realistic RBC flows in the organ-specific morphologies of PCNs.

In the present work, we revisit the original “sheet flow” model and quantify for the first time RBC flows in anatomically inspired *in vitro* models of alveolar capillary networks; our microfluidic platforms recreate at true scale representative organ-specific PCN structures with near-physiological Hct levels (∼33%). To capture experimentally the breadth of RBC dynamics present in such networks, we implement high-speed tracking velocimetry techniques and extract time-resolved Lagrangian data on the instantaneous velocities and ensuing trajectories of RBCs. Together, such measurements deliver ensemble statistics of RBC dynamics directly at the PCN microscale, as a function of pressure drop and the characteristic capillary segment size. To bridge our understanding of local RBC dynamics with bulk flow properties across the entire microfluidic network, we investigate the relationship between volumetric flow rate and pressure drop; this latter strategy permits us to quantify the hydrodynamic resistance of the PCN and its dependence on capillary segment size. By characterizing the underlying transport characteristics of RBCs in anatomically inspired capillary networks under normal physiological conditions, our efforts establish a robust biomimetic *in vitro* platform that offers subsequently a promising gateway for recapitulating and monitoring other physiological and pathological processes occurring directly at the capillary network level.

## II. METHODS

### A. Device fabrication

**FIG. 2.**
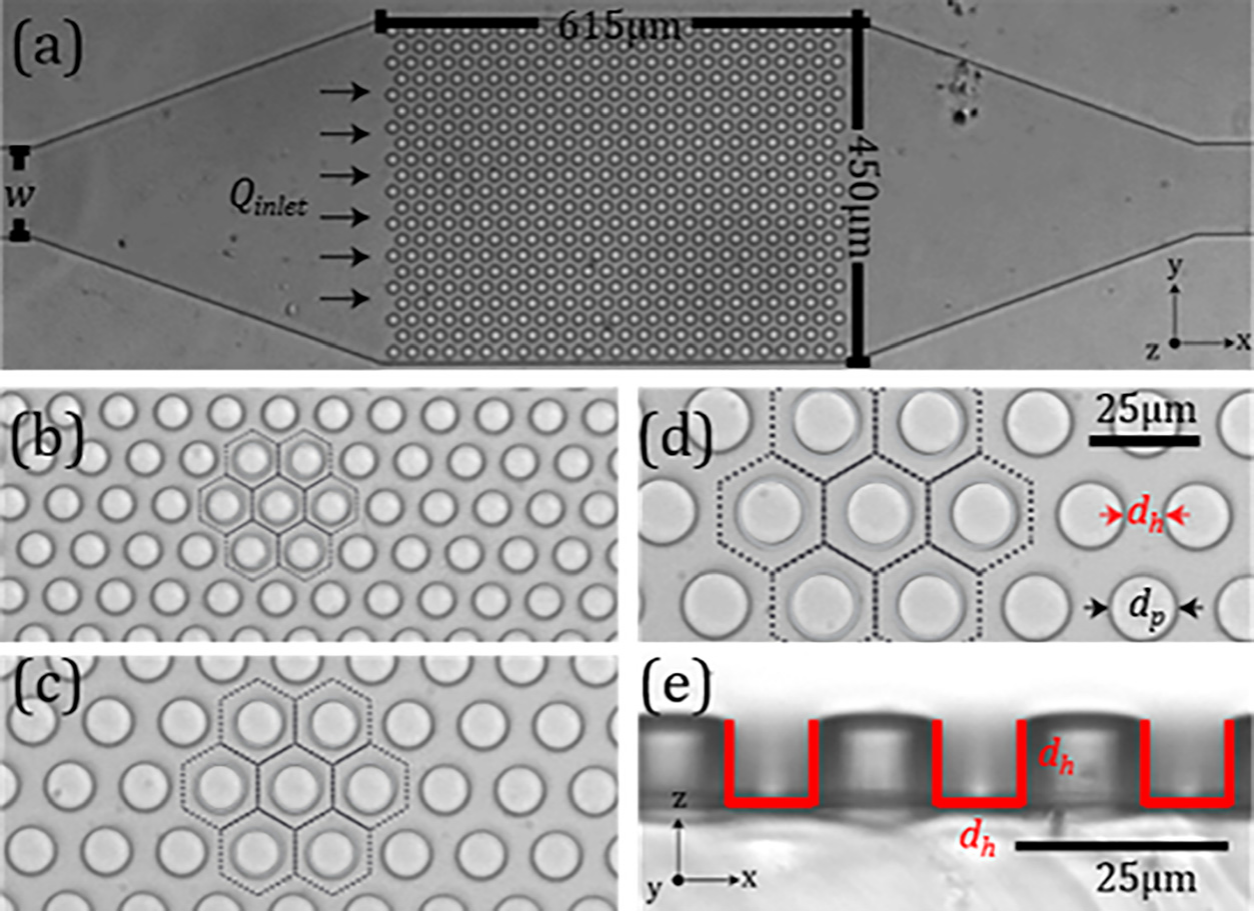
Microfluidic models of the pulmonary capillary networks (PCN). (a) Top view of a microfluidic device. RBC suspensions are perfused from the inlet channel of width *w* (left) across the PCN array and drained at the outlet (right). The PCN mimics the “sheet flow” model and is constructed of a repeating lattice of posts that are arranged as a staggered array across the domain. Note that the inter-distance between two adjacent posts is held constant. Three distinct models were fabricated with an inter-distance between posts of 5 *μ*m (b), 7 *μ*m (c), and 10 *μ*m (d), respectively. For each model, the repeating lattice is highlighted (see hexagon formation). In (d), the inter-distance between posts is marked as *d_h_* while the post diameter is *d_p_*. (e) Cross-sectional view of the *d_h_* = 10 *μ*m model. The height of the model is set to *d_h_* giving rise to a square cross-section between two posts.

Following classic descriptions of the “sheet flow” model,^48^ our microfluidic devices are constructed of a repeating lattice with regularly positioned posts that are spaced by an inter-distance *d_h_* and arranged in a staggered array (Fig. 2). Three distinct microfluidic models were designed with *d_h_* of 5 *μ*m (Fig. 2(b)), 7 *μ*m (Fig. 2(c)), and 10 *μ*m (Fig. 2(d)), respectively. The height of the posts in each geometry is also fixed as *d_h_* such that the local cross-sectional area through which RBCs flow in the *x-z* plane is effectively a square of aspect ratio unity (Fig. 2(e)). Accordingly, the distance *d_h_* corresponds to the characteristic length scale of an individual capillary segment.^48^ The post diameter, *d_p_* (shown in Fig. 2(d)), ranges between ∼3.6 and 7.2 *μ*m such that the ratio *d_h_*/*d_p_* is held constant across each geometry and the net perfused area (i.e., area available for flow) remains fixed at ∼70%. Overall, the PCN geometries cover a surface area representative of ∼2 to 5 alveoli.^55^ The entire array is centered within the microfluidic flow device (Fig. 2(a)), where RBC suspensions (see *Blood preparation* below) are perfused through the domain inlet via a rectangular channel of width *w = *120 *μ*m (and height *d_h_*) that smoothly expands into the network. Past the PCN array, the flow returns to the outlet in a symmetrical fashion where RBCs are drained (Fig. 2(a)).

A standard soft-lithography microfabrication technique for polydimethylsiloxane-based (PDMS) microfluidic devices was adopted,^56^ with a modified method for master production using deep reactive ion etching (DRIE) of a silicon wafer.^57^ This latter method is preferable for the fabrication of features that are 10 *μ*m or less. For the master fabrication, a 4″ Si double-side polished wafer was thermally oxidized to a thickness of 100 nm. The wafer was coated with an AZ 4533 positive tone photoresist to a thickness of approximately 3 *μ*m, pre-exposure baked at 110° C for 2 min, and exposed to UV light using an MA6 mask aligner (Karl Suss) with a dose of 43 mJ·cm^−2^ where the UV light was projected through a chrome mask containing the features of the desired model. After UV exposure, the wafer was developed in TMAH 2.5% for 120 s. The oxide layer was then removed from the areas of the wafer that were not covered by photoresist using Plasma-Therm 790 reactive ion etching (RIE) with CHF_3_ gas. The subsequent DRIE of silicon cavities to the depth of 5 *μ*m, 7 *μ*m, and 10 *μ*m, respectively, was performed in a Plasma-Therm Versaline Inductively Coupled Plasma (ICP) by continuous processing with a gas mixture of SF_6_/C_4_F_8_/Ar. This process results in an anisotropic profile, while avoids scalloped sidewalls.^58^ The residual photoresist was removed in commercial solvents (NMP@75 C and PRS@75 C), followed by Piranha (H_2_SO_4_:H_2_O_2_) solution. Finally, the master was coated with a hydrophobic layer (FTDS) using a molecular vapor deposition (MVD) system to impart anti-stiction properties and ease subsequent PDMS peeling.

For the FDTS ((Heptadecafluoro-1,1,2,2-tetrahydrodecyl) trichlorosilane) deposition process, the Si wafers were put in a MVD100E (Applied microstructure Ltd. USA) vacuum chamber (T = 35 °C, working pressure 20 mTorr). At first, samples were exposed to oxygen plasma at 200 W for 2 min, then the chamber was pumped back to working pressures and 2 cycles of deposition were carried out. The deposition cycle included an introduction of FDTS with water which reacts with the Cl in the silane to form HCl and OH groups that bond to the Si in the silane, CF_3_ (CF_2_)_7_(CH_2_)_2_SiCl_3_ + 3H_2_O → CF_3_ (CF_2_)_7_(CH_2_)_2_Si(OH)_3_ + 3HCl; these OH groups bond to the "open traps" (Si atoms) on the surface and bond to the neighbouring molecules (releasing the Hydrogen) to form stacked self-assembled mono-layer coverage of the surface. Between the deposition cycles, the chamber was purged with N2 to clear the waste products (mostly HCl and water vapor).

Devices were punched using a 23 gauge blunt needle to open the channel inlet/outlet and sealed onto a glass-slide using O_2_.

### B. Blood preparation

Whole blood was taken from healthy human volunteers. Plasma was removed by centrifugation (800 × g for 5 min, 22 °C) and discarded. Pelleted RBCs were re-suspended in 50 ml of phosphate buffered saline (PBS, Sigma, USA); the pH level of the buffer was measured to be 7.16 ± 0.1 using a pH-meter (Mettler Toledo Seven Easy). The suspension was then passed through a leukoreduction filter (RN, Haemonetics, USA). The leukoreduced RBC suspension was washed in PBS (800 × g for 5 min, 25 °C) and adjusted to a 33% haematocrit (Hct) by resuspending the RBCs in buffer. A haematocrit of 33% was maintained across all experiments, as higher Hct levels nearing innate *in vivo* values compromised the adequate tracking of individual RBCs for the given flow conditions and imaging setup. More than 6 samples were used to complete each single experiment described in the work. All procedures were approved for *in vitro* experiments by the Carmel Hospital Institutional Review Board (IRB), Haifa Israel.

### C. Experimental setup

The experimental setup consists of an inverted microscope (Nikon Instruments) mounted with ×40 or ×60 objective lenses and a high-speed CMOS camera (Photron). For each experiment, the pressure drop (ΔP) between the inlet and outlet of the microfluidic device is maintained and controlled by an automated computer-controlled pressure-driven system (Fluigent MFCS-EZ, France), with values ranging between 0.1 and 2 kPa. Such pressure drops give rise to a range of physiologically realistic RBC velocities as measured in pulmonary capillaries *in vivo*^59^ and match the dimensionless Reynolds numbers^9,11^ describing the ratio between inertial and viscous forces (see Section III). RBC suspensions are perfused into each model via a 22 gauge polyethylene tubing system (Instech, USA). Halogen-illuminated images are taken in the mid-plane of the device with a resolution of 1024 × 1024 pixels and frame rates varying between 50 and 2000 fps, depending on pressure drop (0.1–2 kPa) and channel geometry (*d_h_* = 5, 7, or 10 *μ*m), to guarantee adequate tracking of individual cells across all experiments.

### D. Image and data analysis

Image sequences were first processed for optimized detection of RBCs (Davis 8.0.8, LaVision GmbH, Germany). Image backgrounds were removed by subtraction of the average absolute intensity values over the entire sequence, such that moving objects appear brighter than the background. The final grayscale images were converted to binary images upon selection of an optimal threshold. Next, the coordinates of the centre of mass of individual RBCs were detected (Matlab) to track cell trajectories from frame to frame and extract the instantaneous and average velocities of the detected RBCs along their trajectories. For each experiment, more than 2000 individual trajectories spanning at least 100 individual frames were detected and analysed. In parallel, our tracking methods enable us to monitor RBC count in the field of view (FOV). Conducting such cell detection across all experiments (i.e., varying pressure drop and PCN geometry with *d_h_* = 5, 7, and 10 *μ*m) over a span of 100 frames yields a count of 2062 ± 90 RBCs per frame. We note that our cell counting method does not, however, represent a direct estimation of the local haematocrit (Hct) given the imaging and detection limitations in our PCN models (e.g., two-dimensional (2D) imaging projection across a 3D volume; obstruction of RBCs, etc.). Nevertheless, our results for a relatively constant cell count would suggest that with decreasing pillar height (*d_h_*) the cell density increases since the projected area for flow is constant across all PCN models (see Section II A) while the volume of the domain scales linearly with *d_h_*.

Next, two-dimensional (2D) particle image velocimetry (PIV) was used to quantify flow rates, Q_inlet_, at the inlet of the microfluidic device (Fig. 2(a)). Note that PIV measurements are restricted here to the inlet channel only, where the channel width (120 *μ*m) is well within the advocated size range following Poelma *et al*.^60^ and RBCs may thus be directly used as tracers to map velocity profiles across the inlet channel. For each independent experiment, a total of 1000 individual frames was acquired at a frame rate compatible with the applied pressure drop (ΔP). Image sequences were pre-processed (as discussed above) and then analysed using a sum-of-correlation algorithm.^61,62^ The volumetric flow rate across the channel cross-section was approximated as Q_inlet_ = $\bar{v\;}\cdot w\cdot d_{h}$, where $\bar{v}$ is the average velocity across the resulting vector maps, covering a channel area of width *w* (120 *μ*m) and length of 290 *μ*m.

## III. RESULTS AND DISCUSSION

Inspired by the seminal work of Fung and Sobin,^48^ we have aimed to capture in experiments some of the RBC dynamics anticipated to occur within PCN-like structures. We recall that in its original description, the "sheet flow" model^48^ is based on the pulmonary capillary model first introduced by Weibel^63^ where hexagon-shaped lattices were smoothened into circular ones; a feature we have mimicked in our own microfluidic PCN models. In contrast, Fung and Sobin used a scaled-up model constructed at the centimetre scale, where a pressure-driven system was used to flow liquid silicone in the range of Re ∼ 10^−3^–10^−4^. In their experiments, the authors refer to the behaviour of the single-phase fluid (i.e., in the absence of RBCs) and measured experimentally flow-pressure relationships from which a hydrodynamic resistance factor was extracted for various lattice arrangements. In parallel, they investigated using semi-analytical methods streamlines of flow patterns as a function of sheet elasticity. In the footsteps of such efforts, we have measured the dynamics of real RBCs at true scales of the PCN and quantified the ensemble statistics pertaining to velocity magnitudes and lateral displacements (see Sections III A and III B). While our PCN models are static, not unlike Fung and Sobin's experimental efforts, we have measured the pressure-flow relationships for three distinct PCN models (*d_h_* = 5, 7, and 10 *μ*m) from which we extract the corresponding hydrodynamic resistance of the combined RBC suspension and PCN geometry, as discussed in Section III C. Together, these efforts shed new light on the relationship between RBC dynamics at the microscale and bulk flow properties at the scale of the network.

### A. Pathways of RBCs in the capillary networks

**FIG. 3.**
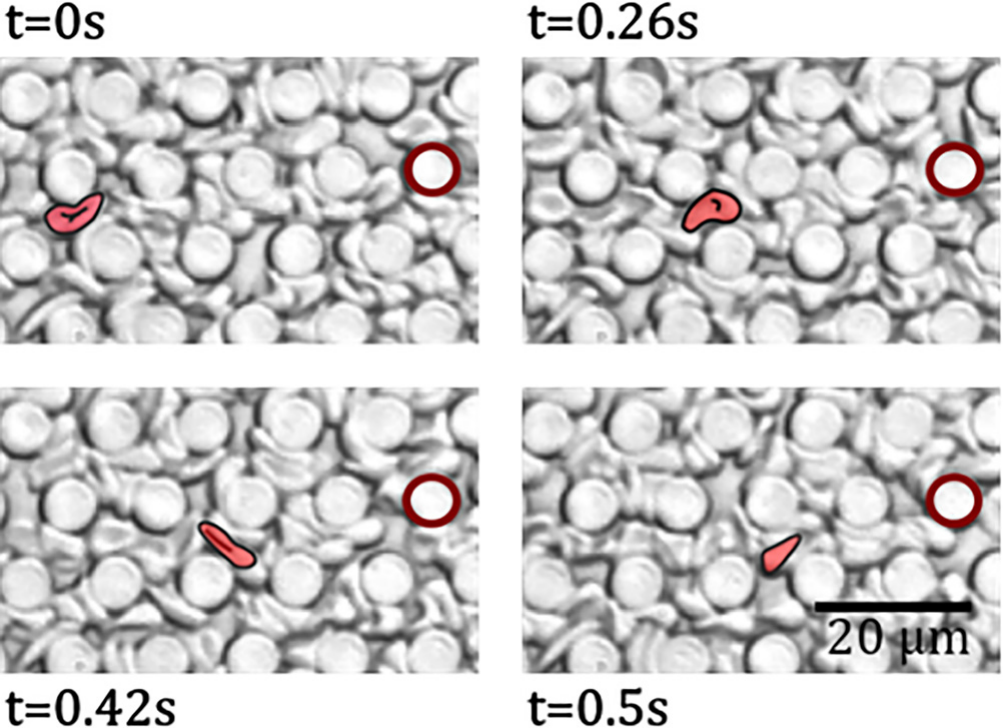
Time lapse sequence of RBCs (Hct = 33%) in a microfluidic PCN model with *d_h_* = 5 *μ*m (ΔP = 0.5 kPa). The labelled cell (shown in red) deforms significantly under constriction from neighbouring cells as it translocates between adjacent posts. Note the non-uniform time steps from one frame to the next; we have marked the contour of a post on the right-hand side to highlight the RBC's translocation in the fixed field of view.

We have conducted RBC tracking velocimetry experiments, and corresponding bulk flow rate measurements (at the device inlet), for a range of pressure drops across each microfluidic PCN model (captured by capillary segment size *d_h_* = 5, 7, and 10 *μ*m), respectively. To first gain qualitative insight into the dynamic flow behaviours of RBCs witnessed in such networks, representative movies are provided in the supplementary material (videos 1–3) for each geometry at a fixed ΔP = 1 kPa. As anticipated with decreasing inter-post spacing *d_h_*, RBCs are increasingly constricted and deform in response; this behaviour is exemplified for a labelled RBC in the time-lapse sequence of Fig. 3 (*d_h_* = 5 *μ*m; ΔP = 0.5 kPa). Note that the instantaneous shape of the highlighted RBC evolves as it makes its way between the posts in the presence of neighbouring cells. In turn, the RBC's instantaneous velocity is intrinsically unsteady, as underlined from the non-uniform time stamps of Fig. 3.

**FIG. 4.**
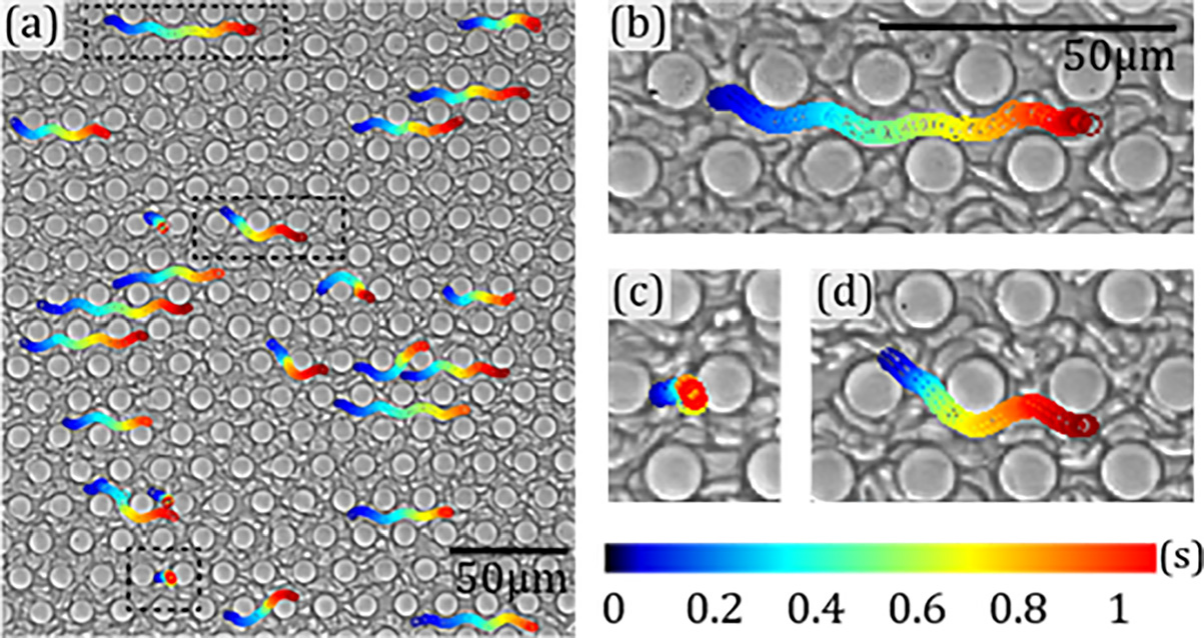
RBC tracking and flow behaviour in microfluidic PCN models. (a) Examples of characteristic RBC trajectories observed in the microfluidic model with *d_h_* = 7 *μ*m (ΔP = 1 kPa); trajectories are color-coded according to time (see colorbar). Three broad behaviours are observed where sample trajectories exemplify (b) RBCs flowing along quasi-straight pathways in the streamwise direction, (c) RBCs trapped between adjacent posts, and (d) RBCs that cross from one axial route to a neighbouring one.

In a next step, we showcase the diversity of RBC pathways observed to coexist synchronously within a single PCN model; this is captured in the representative trajectories of Fig. 4(a) (*d_h_* = 7 *μ*m; ΔP = 1 kPa) that are color-coded according to time and span approximately 1 s (see also supplementary material Video 4). Qualitatively, we distinguish three broad types of trajectories that emerge across the microfluidic PCN models. Recalling that bulk flow arises axially from left to right (Fig. 2(a)), RBCs are first seen to move seamlessly along quasi-straight paths traced between posts following the streamwise direction (Fig. 4(b)). RBCs can instead be locally trapped between two posts that separate the main longitudinal pathways (Fig. 4(c)). In contrast to the rapid transit motions witnessed for the first group, trapped RBCs exhibit little, if any, net translocation over the same fixed time interval. In a third category fitting between these two contrasting transport behaviours, RBCs may exhibit trajectories that cross spanwise from one longitudinal pathway to another, while avoiding complete trapping (Fig. 4(d)).

**FIG. 5.**
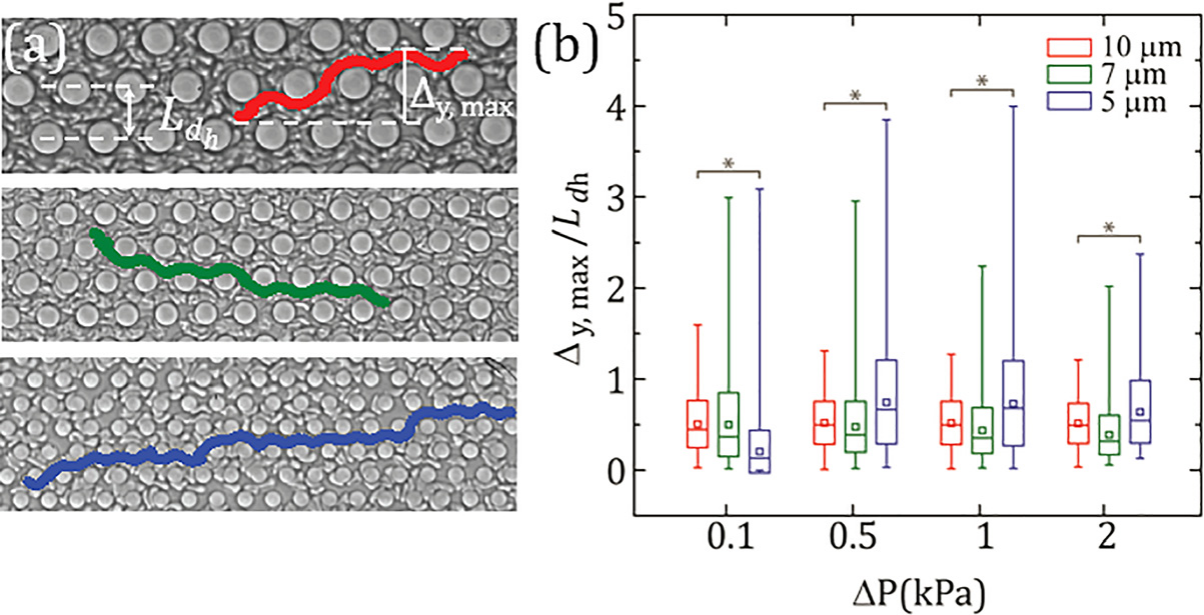
Maximal lateral displacement of RBCs along their trajectory. (a) Example of the maximal lateral displacement, *Δ_y,max_*, travelled by RBCs as observed during tracking across the PCN model; we define a characteristic pathway width, *L_d_*_h_, as the reference distance between the centres of posts lying on adjacent rows. The ratio *Δ_y,max_*/*L_d_*_h_ quantifies the maximum number of pathways a RBC has traversed laterally. In the examples shown (ΔP = 1 kPa) for *d_h_* = 10 *μ*m (red), 7 *μ*m (green), and 5 *μ*m (blue), the ratio yields ∼1.27, ∼2.2, and ∼3.4, respectively. (b) Summary statistics for *Δ_y,max_*/*L_d_*_h_ as a function of pressure drop (ΔP). For convenience, box plots of each PCN model (see legend) are grouped above a fixed ΔP value; each box plot represents over 2000 individual RBC trajectories, where minimum and maximum values of *Δ_y,max_*/*L_d_*_h_, as well as the mean (central line), median (central square), and standard deviation (box boundaries), are shown. Velocity distributions are statistically significant (*p < *0.001) following a Wilcoxon rank sum test that compares pairwise the medians of distributions.

To quantify the occurrence and extent of RBCs crossing laterally from one longitudinal pathway to another (or beyond), we measure the magnitude of the lateral displacement of RBCs along the spanwise *y*-direction. Here, we denote the largest lateral distance that a RBC has travelled along its trajectory as *Δ_y,max_* (see Fig. 5(a)). We compare this maximal lateral displacement to the characteristic longitudinal pathline width, *L_dh_*, defined as the distance between the centres of posts lying on adjacent rows of the PCN and showcased in Fig. 5(a) (top). Note that *L_dh_* thus depends on the characteristic capillary segment size, *d*_h_, such that for *d*_h_ = 10, 7, and 5 *μ*m the value of *L_d_*_h_ is 20.8, 14.6, and 10.4 *μ*m, respectively. In Fig. 5(a), example trajectories of RBCs are shown at ΔP = 1 kPa in the 10 *μ*m (red), 7 *μ*m (green), and 5 *μ*m (blue) PCN models, respectively, where RBCs are shown to cross one or more neighbouring pathways (i.e., the ratio *Δ_y,max_/L_dh_* measured for each is 1.27, 2.2, and 3.4, respectively). Ensemble statistics of the ratio *Δ_y,max_*/*L_dh_* as a function of pressure (ΔP) are summarized in the box plots of Fig. 5(b), for each PCN geometry (see legend), depicting the minimum and maximum values of *Δ_y,max_*/*L_dh_* as well as the median (central line), mean (central square), and standard deviation (SD) (box boundaries).

While statistically significant differences may be observed between the various distributions of *Δ_y,max_*/*L_dh_* (e.g., between 10 and 5 *μ*m, Fig. 5(b)), the general trend remains strikingly similar irrespective of ΔP. Namely, our data support that under the given experimental flow conditions imposed across the pulmonary capillary network (PCN) models, where bulk flow is largely confined along the longitudinal streamwise *x*-direction, both means and medians of the ratio *Δ_y,max_*/*L_dh_* are well below unity. In turn, individual RBC trajectories are widely constrained within a single longitudinal pathline, thus exhibiting relatively little lateral displacement. This is especially true for *d*_h_ = 5 *μ*m where maxima are almost always near unity. With increasing *d*_h_ (e.g., *d*_h_ = 10 *μ*m), and thus a relaxation in RBC confinement, we note the occurrence of few but significant deviations with maximum values of *Δ_y,max_*/*L_dh_* reaching up to ∼3–4.

### B. Ensemble dynamics of RBCs in the PCN

**FIG. 6.**
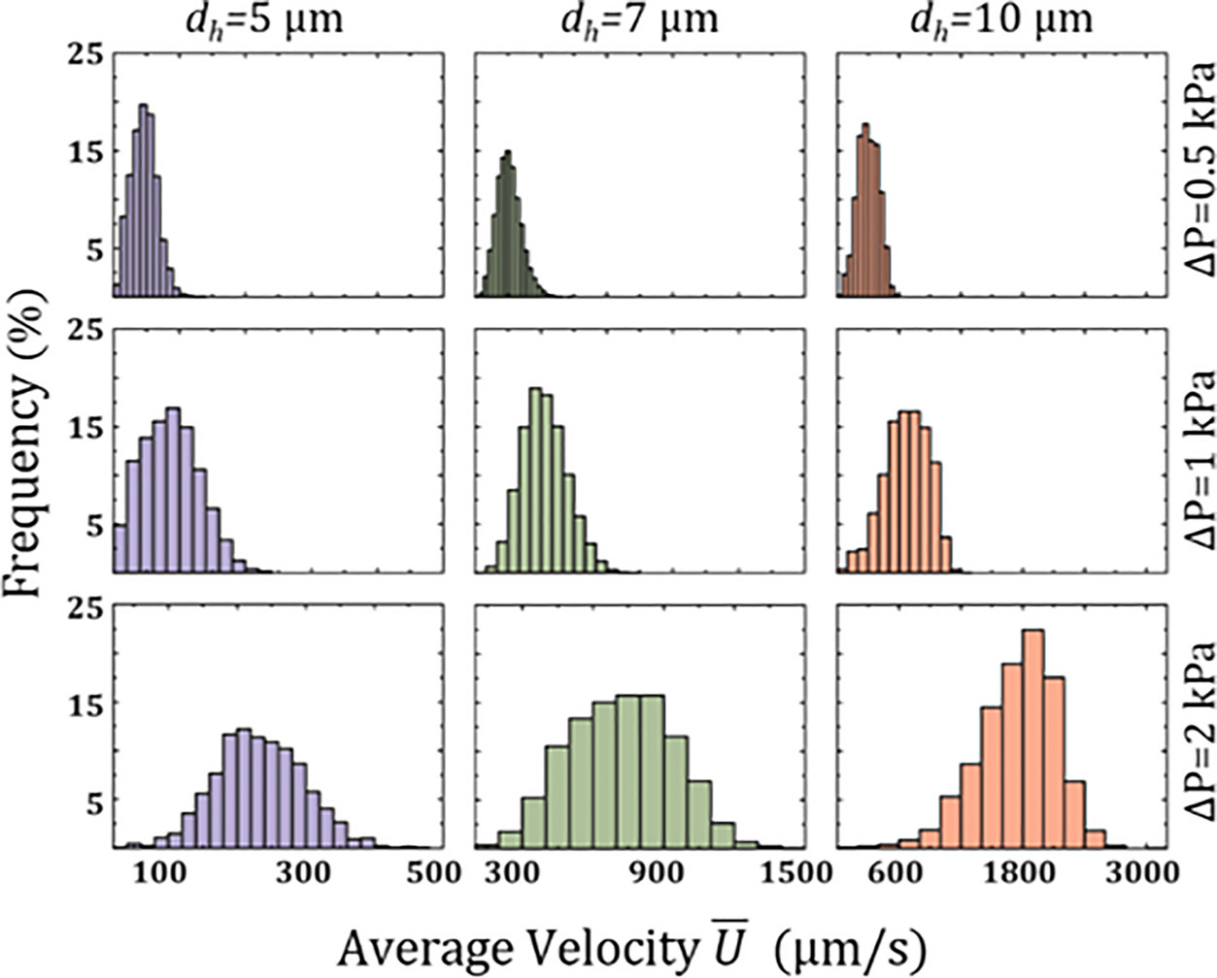
Normalized frequency count for the average RBC velocities ($\bar{U}$) in the microfluidic PCN models, shown as a function of inter-post spacing *d_h_* (columns) and pressure drop ΔP (rows). Each histogram represents over 2000 individual RBC trajectories where Hct is maintained at ∼33% across all experiments. Note the distinct velocity scales (*x*-axis) in each column.

To compare the ensemble dynamics of RBCs across experiments, we calculate the instantaneous (frame-to-frame) velocity of individually tracked cells and extract the average velocity ($\bar{U}$) along their respective trajectory. Quantitative trends for RBC ensembles are presented in the histograms of Fig. 6, where we depict the normalized distributions of $\bar{U}$ as a function of characteristic capillary segment size (*d_h_*) and pressure drop (ΔP). We recall that each histogram represents over 2000 individual trajectories where for conciseness and readability three values of ΔP are shown (i.e., minimum, a mid-value, and maximum), while Hct is maintained at ∼33% across all experiments.

We begin by noting that average velocities of measured RBCs are generally observed to span the range of ∼10^2 ^*μ*m/s, with maxima reaching slightly beyond ∼10^3 ^*μ*m/s (depending on pressure drop and capillary segment size). Such values are compatible with past *in vivo* measurements^59^ of RBCs as well as those obtained with injected microparticles^64^ that reach average velocities ∼500 *μ*m/s. Correspondingly, the values of the Reynolds number for blood flow in capillaries are known to be very low^9,11,65^ and typically fall in the range of ∼10^−4^–10^−3^. In the present experiments, characteristic scales of the local Reynolds number, defined as $Re=\rho \bar{U}d_{h}/\mu$ where ρ and μ are, respectively, the density^66^ (1060 kg/m^3^) and dynamic viscosity^67^ (∼2 × 10^−3 ^Pa s) of blood at Hct levels of 33% diluted in PBS, yield values in the general range of ∼2.6 × 10^−5^ to 9 × 10^−3^, with maxima reaching slightly beyond ∼10^−2^ for RBCs traveling along the fastest pathways. Overall, these values are consistent with the physiological range anticipated in PCNs.

Two overarching characteristics transpire from the velocity data. First, for a fixed *d_h_* (columns of Fig. 6), the span of average velocities ($\bar{U}$) is stretched when pressure drop is increased such that the distributions widen; this result is not surprising when considering the general relationship anticipated between the flow and pressure drop. More markedly, however, the range of average velocities escalates significantly at fixed ΔP (rows of Fig. 6), when *d_h_* gradually increases from 5 to 10 *μ*m; note the velocity scales on the *x*-axis in each column of Fig. 6 that range from 0 to 500 *μ*m/s, 0 to 1500 *μ*m/s, and 0 to 3000 *μ*m/s, respectively. In other words, with increasing inter-post spacing RBCs can flow more freely at a given pressure drop; a likely consequence of the lesser constrictions imposed by wider spacing (supplementary material Videos 1–3). Interestingly, all the histograms of Fig. 6 point to the existence of two persistent "tails" in the velocity distributions: the left ones with near-zero values of $\bar{U}$ capture the behavior of slow moving RBCs that underline mostly trajectories of trapped cells (Fig. 4(c)). In contrast, the right tails describe the fastest moving RBCs that travel undisturbed along streamwise pathways. Since the frequency of events on either tails of the distributions remains altogether very low, the majority (i.e., mean and standard deviation) of tracked RBCs are expected to exhibit transport dynamics with features lying in between trapped and free motions (Fig. 4(d)).

**FIG. 7.**
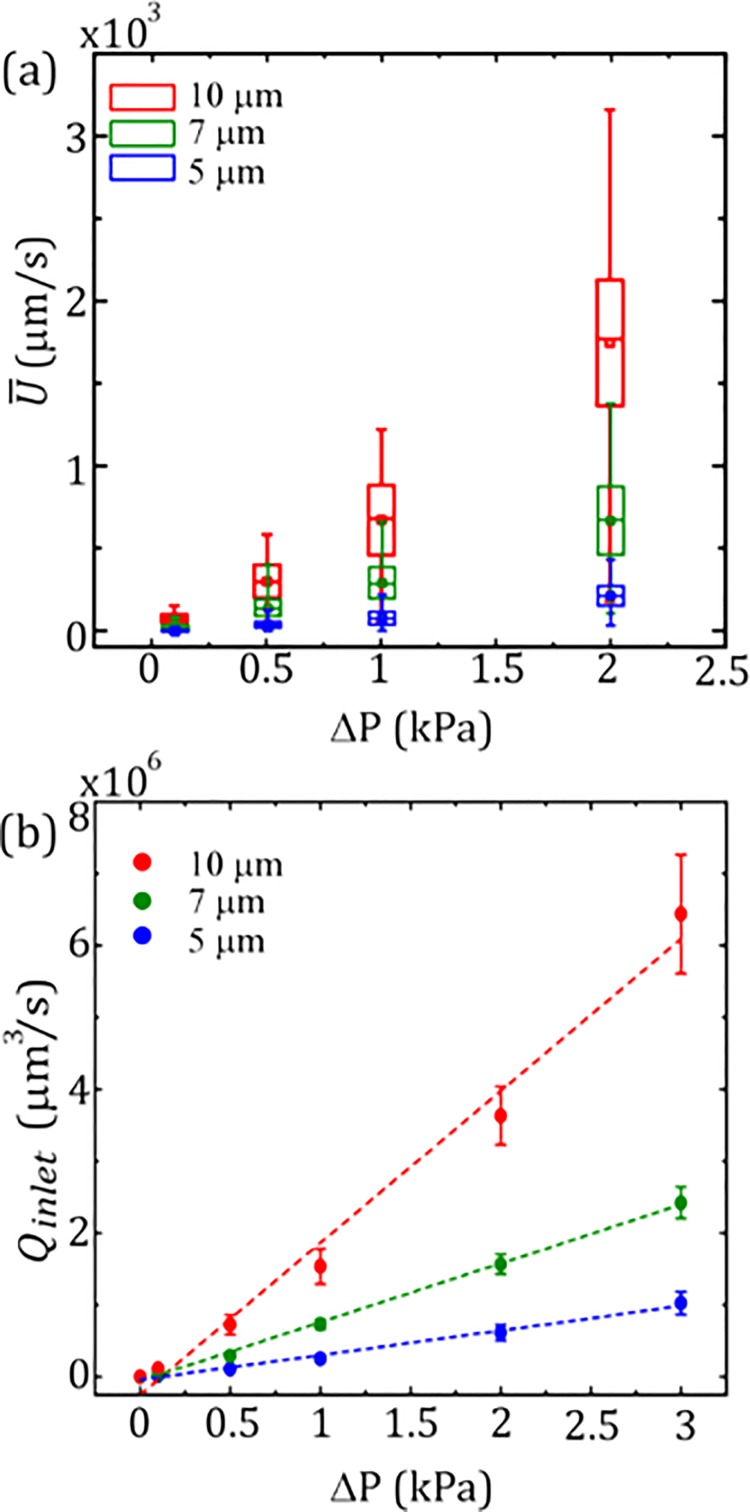
RBC velocity magnitudes and Flow-pressure relationships in the PCN models. (a) Average RBC velocities ($\bar{U}$) as a function pressure drop (ΔP). Each box plot exhibits minimum and maximum values of $\bar{U}$, as well as the mean, median, and standard deviation. Velocity distributions are found to be statistically significant (*p < *0.01) following a Wilcoxon rank sum test that compares pairwise the medians of distributions. (b) Bulk flow measurements ($Q_{inlet}$) across the microfluidic devices as a function of pressure drop (ΔP), where ΔP = 0 corresponds to quiescent flow. Data points (mean +/ SD) represent *n* = 7 independent measurements and are statistically significant with *p* < 0.01 following a Wilcoxon rank sum test. Linear regressions yield r^2 ^= 0.986, 0.998, and 0.991 for *d_h_* = 10, 7, and 5 *μ*m, respectively. Note that the inverse of the slopes corresponds to the hydrodynamic resistance (R) of each PCN model (see text for details).

The velocity distributions of Fig. 6 are quantitatively summarized in the box plots of Fig. 7(a) that depict the minimum and maximum values of the average RBC velocities, as well as the median, mean, and SD. Following the discussion above, a positive monotonic trend emerges for $\bar{U}$ as a function of ΔP for each fixed capillary segment size. While the velocity distributions are found to be statistically significant (*p < *0.01) following a Wilcoxon rank sum test that compares pairwise the medians of distributions, we note that the values of $\bar{U}$ between the edges of the box plot (i.e., SD), and in particular, the whiskers (i.e., minimal and maximal values), may overlap when comparing a fixed pressure drop. This phenomenon is in line with the observation across all experiments that there are always RBCs that are eventually trapped. While these events become increasingly seldom with larger ΔP and wider inter-post spacing, our data emphasize nevertheless how RBC transport in the PCN models is intrinsically heterogeneous and dynamic.

### C. Network resistance of the PCN: Bulk flow versus pressure drop

The positive correlation observed in Fig. 7(a) between $\bar{U}$ and ΔP gives a first glimpse into the hydrodynamic resistance of the PCN model as a function of *d_h_* in the presence of flowing RBCs. We anticipate that the total resistance of the network to blood flow is influenced by both the intrinsic properties of blood and the geometric features of the domain in which RBCs flow. We recall that the geometrical changes operated between the distinct geometries, beyond modifying the inter-post spacing, are anticipated to be negligible; namely, the projected area available for flow is held constant across all devices (∼70%) since the ratio of *d_h_*/*d_p_* is fixed (see Section II). Furthermore, the ratio between the total wetted area for *d_h_* = 10 *μ*m and 7 *μ*m compared to 5 *μ*m is 1.01 and 0.994, respectively. To a first approximation, the variable anticipated to influence the overall hydrodynamic resistance of the network is the capillary segment length *d_h_*; this parameter captures the necessity for RBCs to deform under confinement. In analogy, Obrist *et al*.^53^ introduced the notion of an effective resistance combining the resistance of the capillary according to Poiseuille's law with an additional resistance due to the presence of RBCs; the latter directly correlated with the capillary diameter. In turn, with decreasing capillary segment size the resistance would increase in both elements composing the effective resistance.

To quantify the hydrodynamic resistance in the respective networks, Fig. 7(b) presents measurements of the bulk flow rate ($Q_{inlet}$) across the microfluidic devices (see Section II) as a function of ΔP. Here, data points (mean +/ SD) represent *n* = 7 independent measurements and are all found to be statistically significant with *p* < 0.01 following a Wilcoxon rank sum test. Recalling that the PCN models are complex networks that deviate significantly from simple, straight tubes, a first striking observation is the near linear behaviour of the flow rate as a function of pressure drop in each respective micro-device. Such trends are well captured by a linear regression with r^2 ^= 0.98, 0.99, and 0.99 for *d_h_* = 10, 7, and 5 *μ*m, respectively. Note that in the present experiments, we have extended the range of imposed ΔP (max. of 3 kPa) beyond the maximal pressure drop where single cell tracking is still guaranteed with the given imaging setup.

Utilizing the general relationship (e.g., Poiseuille law) $\;\Delta P=Q_{inlet}R$, we may extract the hydrodynamic resistance (R) of each PCN from the inverse of the slopes of Fig. 7(b). Here, we find *R* = 0.47, 1.22, and 2.92 mPa·s/*μ*m^3^ for *d_h_* = 10, 7, and 5 *μ*m, respectively. These values underline how the hydrodynamic resistance of the network increases monotonically with decreasing inter-post spacing. The linear correlation observed here between the flow rate and pressure drop across PCNs was first reported in the “sheet flow” model,^48^ using scaled-up experiments in the absence of RBCs. The authors noted then that the presence of posts was indeed very effective in increasing hydrodynamic resistance. Yet, these seminal efforts came short of characterizing RBCs: their presence is known to affect hydrodynamic resistance and apparent viscosity ($\mu_{app}$), as demonstrated in microvascular networks *in vivo*.^7,68^

Unlike a single-phase Newtonian fluid in a vessel obeying Poiseuille flow with $R=8\mu L/\pi r^{4}$ and a fixed dynamic viscosity μ, we cannot discriminate how changes in R are determined by the PCN geometry (i.e., *d_h_*) versus the apparent blood viscosity ($\mu_{app}$); our experiments do not directly quantify the functional relationship $R=f\left(d_{h},\mu_{app}\right)$. Despite such limitations, we may draw attention to the fact that the relative change between the resistance of the PCN with *d_h_* = 10 *μ*m and 5 *μ*m, i.e., *R*_10_ and *R*_5_, is $\left|R_{5}-R_{10}\right|/R_{5}=0.83\;$ while the relative change between the capillary segment *d_h_* = 10 *μ*m and 5 *μ*m is 1. Correspondingly, the relative change between *R_7_* and *R_5_* and capillary segments *d_h_* = 7 *μ*m and 5* μ*m is 0.58 and 0.40, respectively. Taken together, our results suggest the existence of a direct functional relationship between the characteristic capillary segment size and the hydrodynamic resistance of the capillary network to blood flow.

## IV. CONCLUSIONS

Following the seminal descriptions of the capillary “sheet flow” model, we have explored the dynamics of RBCs in *in vitro* microfluidic platforms mimicking organ-specific PCN structures. Undoubtedly, these models remain mere simplifications of real alveolar capillary networks, where our experiments are limited to idealized networks with homogenous distributions of pillars and fixed capillary segment sizes. Indeed, the alveolar capillary environment is known to be anatomically heterogeneous;^46^ this latter aspect remains though beyond the scope of the present study and will be addressed in future work. In addition, we have limited our working fluid to RBC suspensions and discarded for instance white blood cells (WBCs), platelets, and plasma. Indeed, WBCs (in spite of their small presence in whole blood by volume, i.e., <1% v/v) may block pathways due to their large size, (∼8–15 *μ*m),^69^ while plasma proteins (e.g., fibrin) and platelets may obstruct pathways via thrombosis,^70^ which would lead amongst other to an increase in the local resistance of the network as well as RBC rerouting phenomena.^52,71^ Moreover, our fabricated models are void of endothelial cells^72^ and remain static with stiff walls lacking the ability for capillary recruitment.^73^ In particular, it is known that alveoli stretch considerably during lung inflation; a property that in turn affects the curvature and thickness of the pulmonary sheet.^48,74,75^ While the long axial pathway lengths travelled by RBCs in the idealized microfluidic network (Fig. 4(a)) are not anticipated to capture the innate physiological milieu as cells are likely to encounter a venule sooner and thus be drained,^47^ the velocimetry data presented (Fig. 6) are in line with *in vivo* velocity measurements of RBCs using intravital microscopy.^59,64^

With such limitations in mind our *in vitro* efforts constitute to the best of our knowledge a first, yet important step in investigating systematically the transport dynamics of RBCs in alveolar capillaries at true scale. Most strikingly, our experiments provide new quantitative insight that bridges between the local dynamics of RBCs and the macroscopic bulk flow properties within organ-specific capillary networks.

## SUPPLEMENTARY MATERIAL

See supplementary material for representative movies (supplementary material Movies 1–4) of dynamic flow behaviours of RBCs in PCNs.
